# Limited doses of immunotherapy use in advanced non‐small cell lung cancer elderly patients with ECOG of 2 and high PDL‐1 expression

**DOI:** 10.1002/agm2.12179

**Published:** 2021-10-24

**Authors:** Pui Yee Wong, Soon Hin How, Radhiana Hassan, Muhammad Naimmuddin Abdul Azih

**Affiliations:** ^1^ Department of Respiratory Medicine Hospital Tengku Ampuan Afzan Kuantan Malaysia; ^2^ Department of Medicine Kulliyyah of Medicine International Islamic University Malaysia Kuantan Malaysia; ^3^ Department of Radiology Kulliyyah of Medicine International Islamic University Malaysia Kuantan Malaysia; ^4^ Department of Medicine Kulliyyah of Medicine International Islamic University Malaysia Kuantan Malaysia

**Keywords:** durable response, high PD‐L1, immunotherapy, poor ECOG, short duration

## Abstract

Immunotherapy is an effective treatment in advanced non‐small cell lung cancer (NSCLC) patients with high PDL‐1 expression. Here, we report three such patients with durable response despite limited doses immunotherapy.

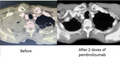

## 1 INTRODUCTION

The treatment landscape of lung cancer has evolved over the years; the standard of care has shifted from chemotherapy to the use of targeted therapy or immunotherapy (IO) as first‐line treatment in patients with advanced non‐small cell lung cancer (NSCLC). Breakthroughs were seen in 2015 and 2016 when IO demonstrated significant survival benefit compared with platinum‐based chemotherapy in previously treated advanced NSCLC. The use of IO as first‐line monotherapy in patients with positive PD‐L1 expression was subsequently approved by the US Food and Drug Administration.^1^, ^2^, ^3^

In randomized clinical trials, treatment with pembrolizumab resulted in significant improvements in progression‐free survival (PFS) and overall survival (OS) compared with chemotherapy alone. An updated analysis of the KEYNOTE‐024 study comparing the outcomes of pembrolizumab versus platinum‐based chemotherapy in treatment‐naïve patients with PD‐L1‐positive advanced NSCLC (TPS of ≥50%) showed that the median OS was doubled following pembrolizumab treatment compared with chemotherapy (30.0 months vs 14.2 months, respectively). Fifty‐four percent of those randomly assigned to chemotherapy crossed over to the pembrolizumab arm and despite that, the improvement in OS from the first analysis was maintained.^2^ In the KEYNOTE‐042 phase III study in NSCLC patients with positive PD‐L1 expression, those assigned to pembrolizumab as first‐line treatment had better OS compared with those on chemotherapy.^4^ The IMpower 110 trial in treatment‐naïve metastatic NSCLC patients with positive PD‐L1 expression showed that treatment with atezolizumab resulted in significantly longer median OS and PFS compared with chemotherapy.^5^

Despite their significant benefits, IO drugs are, nonetheless, expensive and not funded by the national health in most developing countries. Here, we report on three cases of advanced NSCLC with high PD‐L1 expression in the absence of EGFR and ALK alterations. The three patients who were treated with limited doses of IO showed durable partial response for more than 19 months after treatment cessation.

## 2 Case Report

### 2.1 Case 1

In October 2019, an 88‐year‐old lady who is an ex‐smoker of 15 pack‐years with underlying left ischaemic stroke with no residual hemiparesis presented with bilateral lower limb weakness secondary to statin‐induced myopathy. She was referred for an incidental chest x‐ray finding of a left lower lobe mass. She had no shortness of breath, cough, or hemoptysis but appeared lethargic and emaciated. Vital signs were normal. There was dullness on percussion and reduced air entry on the left lower lobe with no palpable supraclavicular lymph nodes. Neurological examination was unremarkable after the statin was discontinued. Blood investigations were normal.

**FIGURE 1:**
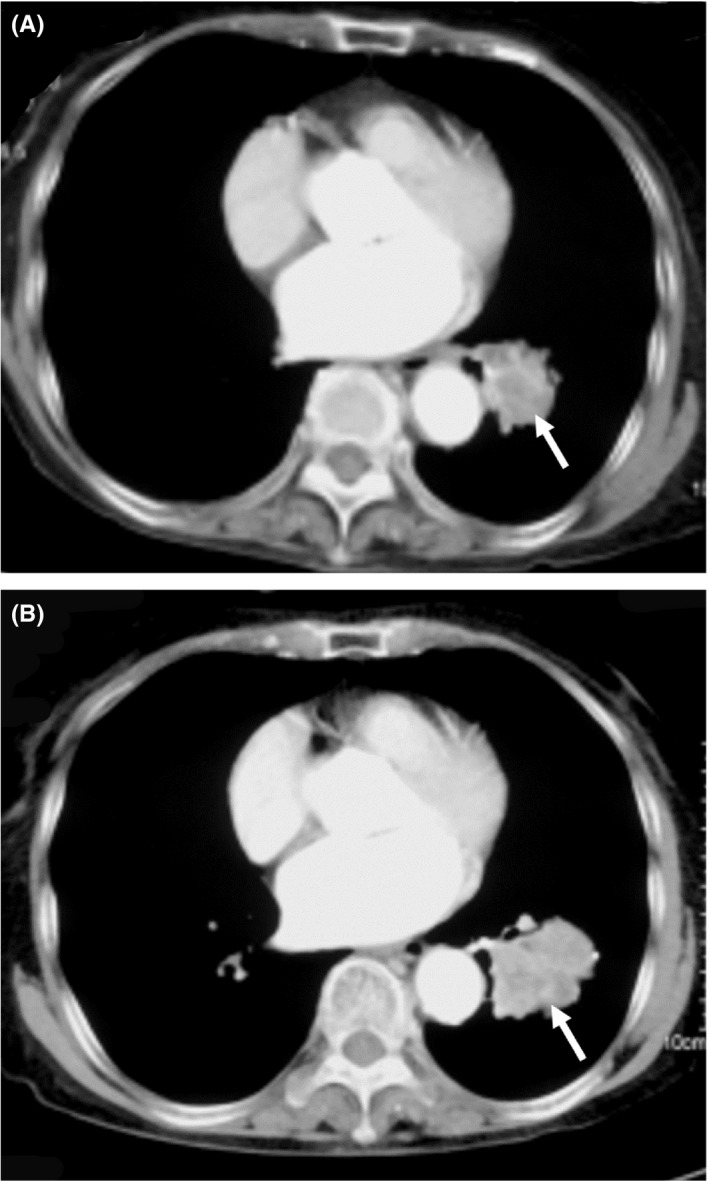
(A) The initial contrast‐enhanced axial plane CT thorax in the soft tissue window in September 2019 showed a tumor mass (white arrow). (B) The size of the tumor mass (white arrow) was reduced 16 months later, compared with the initial scan.

The incidental lung mass was better visualized on a computed tomography (CT) thorax, which showed a heterogenous mass measuring 4.4 × 3.8 × 4.2 cm in the apical segment of the left lower lobe and an enlarged ipsilateral mediastinal lymph node (Figure 1A). CT‐guided biopsy performed with pathological examination of lung squamous cell carcinoma and staging based on the TNM system indicated that the patient had stage 3A NSCLC (T2N2M0). The patient's ECOG status was 2, with no EGFR and ALK mutation detected but she had high PD‐L1 expression of 80%. The patient was not fit for surgery and was not keen for chemotherapy or radiotherapy for fear of the side effects. Owing to financial constraint, the patient agreed to have only two doses of atezolizumab 1200 mg IV administered in March and November 2020. Repeated CT thorax in February 2021 showed good radiological response with the primary tumor size reduced to 2.7 × 2.9 × 2.9 cm (Figure 1B). The patient had good appetite after two doses of atezolizumab. As the patient could afford to pay for another dose, the third dose was administered in February 2021. The patient continued to have good partial response based on response evaluation criteria in solid tumours (RECIST 1.1) and she remained asymptomatic for over 19 months, without developing any adverse reactions, despite having received only three doses of atezolizumab.

### 2.2 Case 2

**FIGURE 2:**
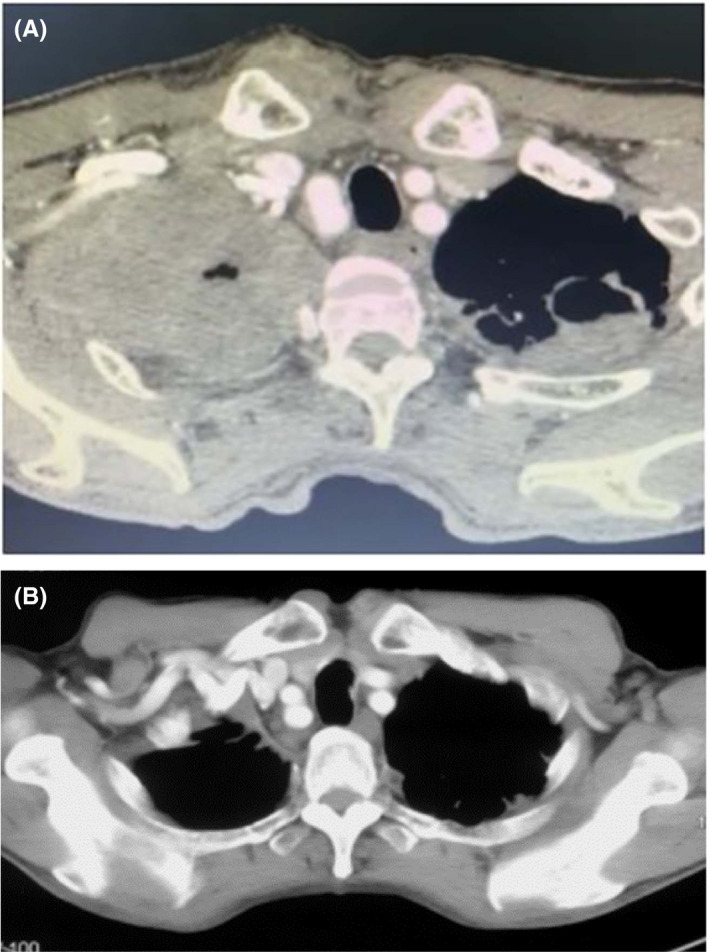
(A) The initial contrast‐enhanced CT thorax in September 2019 showed a large invasive tumor of the right upper lobe with regional bony destruction. (B) A CT thorax in October 2020 showed near complete resolution

An 82‐year‐old gentleman with underlying hypertension and an 80‐pack‐year smoking history presented with persistent right upper back pain, which he had been experiencing for 2 weeks. He had progressive swelling of the right upper chest wall, associated with loss of appetite and a 3‐kg weight loss over a period of 2 months. He denied having shortness of breath, fever, or hemoptysis. Vitals were normal; however, the right supraclavicular lymph node was palpable and a hard, nonerythematous, nontender mass of irregular margin was noted over the right upper chest wall. There was reduced air entry over the right upper lobe. Bloods investigations revealed raised CEA of 580 ng/ml. A CT thorax, abdomen, and pelvis revealed a large tumor measuring 12.4 × 10.0 × 8.4 cm at the apical region of the right upper lobe, fibrotic scarring, and subpleural bullae in the apex of the left upper lobe with areas of cavitation, and enlarged right supraclavicular and multiple mediastinal lymph nodes with a bulky left adrenal gland (Figure 2A). A core biopsy of the right supraclavicular lymph node was performed. The patient was subsequently diagnosed with stage 4A lung adenocarcinoma (T4N3M1b) and ECOG 2. He had high PD‐L1 expression (TPS 95%) and wild‐type EGFR and ALK. The patient was keen on having IO but could not afford to pay for the full recommended course of pembrolizumab (35 doses), which required administration every three weeks (Q3W).

After receiving the first dose of pembrolizumab 100 mg IV in November 2019, the patient's pain was greatly reduced and his serum CEA level dropped significantly to 106 ng/ml. Six weeks later, a second dose of pembrolizumab 100 mg IV was administered without the patient developing significant adverse reactions. A subsequent CT scan in March 2020 showed significant tumor reduction with an apical mass thickness measuring 2.0 cm, minimal left apical pleural thickening, and the previously seen lymph nodes had shrunk to subcentimeters. The patient refused further doses of pembrolizumab because of financial constraint. In October 2020, a CT scan showed similar findings to the CT scan in March 2020 (Figure 2B). The patient had persistent good partial response to pembrolizumab with repeated blood tests showing serum CEA levels of 3.0 ng/ml. At the time of writing this report, the patient has remained asymptomatic with no further weight loss for 19 months.

### 2.3 Case 3

**FIGURE 3:**
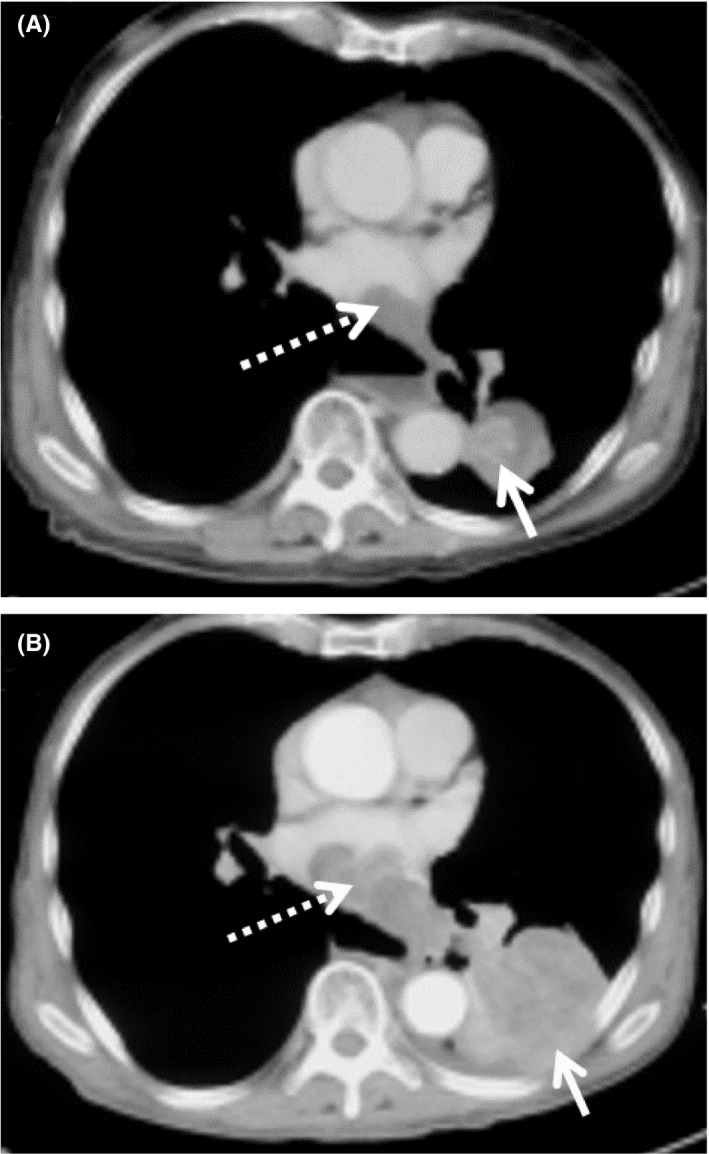
(A) The initial contrast‐enhanced axial plane CT thorax in the soft tissue window–CT thorax in September 2019 showed a mass in the left lower lobe encasing the descending aorta, with tumor thrombus seen in the left atrium. (B) Sixteen months later, the mass in the left lower lobe (white arrow) and the tumor thrombus in the left atrium (dashed arrows) had reduced in size

An 87‐year‐old gentleman, a nonsmoker with underlying hypertension and hyperlipidemia, was referred for an incidental chest x‐ray finding of a mediastinal mass during a health screening. The patient reported experiencing some weight loss but had no episodes of fever, cough, hemoptysis, shortness of breath, or loss of appetite. Neither clubbing nor palpable lymph nodes were observed. However, reduced air entry over the right lower lobe with dullness on percussion was noted. Vital signs and blood investigations were normal. Transthoracic ultrasound‐guided trucut biopsy of the mass was performed and it was confirmed to be adenocarcinoma. EGFR mutation and ALK rearrangement were not detected but the patient was PD‐L1‐positive (TPS 80%). An initial CT scan suggested that the patient had stage 4A lung adenocarcinoma (T4N2M1b) with a mass seen in the left lower lobe measuring 13.3 × 8.8 cm, encasing the descending aorta and extending to the lower oesophagus. Tumor thrombus was seen in the left segmental pulmonary veins extending to the left atrium. Ipsilateral hilar lymph node enlargement and multiple hypodense liver lesions were observed (Figure 3A). The patient's ECOG status was 2.

In November 2019, the patient was given only two doses of pembrolizumab 100 mg IV Q3W owing to financial constraint. The patient gained 3 kg after the first dose without developing side effects. Three months later, a restaging CT scan showed reduction of mass by ≥50% (measuring 4.6 × 4.6 × 4.3 cm), which indicated good partial response to pembrolizumab. The patient agreed to have follow‐up CT scans every 3 months. The radiological findings were stable until September 2020, when a CT scan revealed a slightly increased tumor size of 5.6 × 4.1 × 4.6 cm. The patient agreed to have two more doses of pembrolizumab 100 mg IV given Q3W in October 2020. No adverse reaction was observed. A repeated CT scan showed a reduced mass measuring 4.8 × 3.7 × 4.0 cm and tumor thrombus with stable liver lesions (Figure 3B). The patient remained in good condition with durable partial response for 19 months.

## 3 DISCUSSION

The patients described in these case reports are elderly (i.e. above 80 years of age) with poor ECOG and yet they experienced good response with noncontinuous IO. It is worth noting that two of the patients were given only half the recommended dose of pembrolizumab. None of them had any documented adverse reactions owing to the limited duration of immunotherapy. Chatterjee et al. analyzed data from the Keynote‐001 study of multiple cohorts of treatment‐naïve and previously treated NSCLC who were given different doses of pembrolizumab at 2 mg/kg Q3W, 10 mg/kg Q3W, and 10 mg/kg every two weeks (Q2W). Similar overall response rate (ORR) and disease control rate (DCR) were observed in the 2 and 10 mg/kg doses.^6^ According to our local Malaysian guideline, pembrolizumab can be given at 2 mg/kg Q3W in the second‐line setting. Despite being given pembrolizumab 2 mg/kg, our patients demonstrated durable partial response without disease progression for more than 19 months. As most clinical trials had been conducted for the recommended 2‐year duration, little is known about the optimum duration and clinical benefits of short‐duration IO.

Yilmaz and Guven Mese described two advanced NSCLC patients treated with a short course of nivolumab as second‐line treatment in view of financial constraint. In the first case, the patient had durable complete response with only 9 months of IO treatment and no disease progression for more than 18 months. Another patient discontinued after 4 months of nivolumab and showed complete response for 6 months. Both patients remained in remission for 3 years after the manuscript was published.^7^ ^(e−mail communication with author)^ Babak et al. documented a patient with metastatic lung adenocarcinoma who had complete remission with nivolumab given as fifth‐line treatment, despite having discontinued treatment after 4 months owing to grade 2 transaminitis. The patient remained in complete remission for 14 months.^8^

A study was conducted in Oulu University Hospital involving 59 patients with different tumor types including lung cancer, who were treated with IO following the institutional recommended maximum duration of 6 months. All of the lung cancer patients were given IO as second‐line therapy or more, and the OS was similar to clinical trial data.^9^ The authors observed exceptional durable response in those who had IO for only a short duration, similar to our patients' clinical experience. Our patients could not afford the cost of 35 doses of IO, which would amount between USD75,000 to USD150,000 and is not financed by our institutions. With the average Malaysians' median income of USD1468, many would not be able to afford the expensive cost of IO.^10^

A study of real‐world data conducted by Youn et al. analyzed the prognosis of 1256 patients with a median age of 75.3 years. Almost half of the cohort had multiple comorbidities and 11.5% had poor ECOG status. Half of the cohort received IO after platinum chemotherapy while only 8.1% were treatment‐naïve patients. The median survival after IO treatment was 9.3 months, which could be explained by the shorter survival rate of elderly patients with multiple comorbid conditions.^11^ Our patients, on the other hand, who were above 80 years old, with high PD‐L1 expression and preexisting comorbidities, survived for more than 19 months since being treated with IO.

Gridelli et al. discussed the use of gemcitabine as single‐agent chemotherapy in elderly patients aged more than 70 years with advanced NSCLC. The ORR, based on several phase II trials, was reported as 18% to 38% with a median OS of 6.8–9 months, which were inferior to the outcomes reported in IO clinical trials. A phase II trial of paclitaxel given Q3W in two cohorts of patients below and above 70 years of age showed no difference in the ORR or median OS between the two groups; however, it was noted that the frequency of adverse effects such as neutropenia and toxicity was more prevalent in older patients.^12^ Our patients were not keen on chemotherapy given its known side effects. IO was given instead, in short duration, and none of the patients developed any adverse events.

Most phase III clinical trials exclude patients with poor ECOG (≥2) due to preexisting comorbidities, contraindication to ongoing treatment, potentially lower drug metabolism, and less tolerability to treatment.^13^ Thus, the efficacy of IO as first‐line treatment is not widely known among this group of patients. A multicenter retrospective analysis of 234 metastatic NSCLC patients with PD‐L1 ≥50%, given pembrolizumab as first‐line treatment, showed shorter median OS with subtle difference in the median PFS in patients with ECOG 2 (n = 39) compared with those with ECOG 0–1 (median OS: 7.4 months vs 20.3 months, respectively; median PFS: 4.0 months vs 6.6 months, respectively). The ORR was much lower in patients with ECOG 2 compared with patients with ECOG 0–1 (25.6% vs 43.1%, respectively). However, 28.3% of the ECOG 2 group continued pembrolizumab without disease progression.^3^ These findings were supported by Middleton et al. who conducted a multicenter, single‐arm phase II trial (PePS2) of pembrolizumab Q3W in patients with advanced NSCLC with ECOG 2. Similarly, a median PFS of 4.4 months and a median OS of 9.8 months were observed and both improved with increasing TPS. Durable response was shown to be better with higher TPS level (53% in TPS ≥50% vs 22% in TPS <1%). Among the 60 patients with a median age of 72 years, only 10% discontinued therapy owing to drug toxicity while 15% stopped treatment because of high‐grade adverse events. No fatal treatment‐related adverse events were reported and none discontinued owing to death from hyperprogression.^14^ These studies suggest that pembrolizumab, with its relatively favorable adverse event profile, is safe to be administered in patients with ECOG 2.

## 4 CONCLUSION

We have here reported a case series of three advanced NSCLC patients who received only a few doses of IO and experienced durable partial response for over 19 months despite having poor ECOG and being elderly. Limited doses of IO may be beneficial in patients with high PD‐L1 expression especially in resource‐limited countries.

## CONFLICT OF INTEREST

The authors have no conflicts of interest to declare.

## AUTHOR CONTRIBUTIONS

Pui Yee Wong and Soon Hin How conceptualized the topic, performed literature search, and wrote the case series. Radhiana Hassan analyzed the radiological images as well as reviewed and edited the case series. Muhammad Naimmuddin Abdul Azih reviewed and edited the case series.

## ETHICAL APPROVAL

The authors have obtained written informed consent for publication of patient information and radiological images. In our local setting, IRB approval is not required for publication of these case reports.
